# A Daily Diary Study on Sleep Quality and Procrastination at Work: The Moderating Role of Trait Self-Control

**DOI:** 10.3389/fpsyg.2018.02029

**Published:** 2018-11-02

**Authors:** Wendelien van Eerde, Merlijn Venus

**Affiliations:** Amsterdam Business School, University of Amsterdam, Amsterdam, Netherlands

**Keywords:** procrastination, sleep quality, self-control, employees, diary study

## Abstract

**Background:** This daily diary study investigates the relation between sleep quality during the night and its effect on procrastination at work during the next workday. Previous research has shown that sleep quality is an important variable for work behavior at the daily level, including employee performance, safety, health, and attitudes, such as work engagement. Also, sleep quality has been found to be negatively related to next-day work procrastination. However, these studies did not address trait differences that may be involved. In other words, they have not investigated whether all employees experience the effects of sleep quality on procrastination similarly. We explore the moderating effect of trait self-control.

**Methods:** Seventy one full-time employees (51% male) working in various industries participated, including finance or banking (17%), government or education (13%), construction (7%), health care (7%), sales or marketing (6%), and others. Average age was 35.20 years (*SD* = 12.74), and average employment tenure was 13.3 years (*SD* = 13.16). Participants completed a one-shot general electronic questionnaire (to assess trait self-control, using a four-item scale adapted from Tangney et al., 2004). Subsequently, these employees received two daily electronic questionnaires to assess sleep quality (measured with one item from the Pittsburgh Sleep Quality Index (Buysse et al., 1989), and a three-item scale of procrastination (adapted from Tuckman, 1991) over the course of 10 workdays, resulting in 465 pairs of matched morning-afternoon measurements (65% response).

**Results:** Results of multilevel regression analyses showed that sleep quality was negatively related to work procrastination the next day. Sleep quality, however, also interacted with trait self-control in impacting work procrastination, such that low sleep quality affected employees low in trait self-control, but not employees high in trait self-control.

**Conclusion:** The findings of this study qualify earlier research showing the relation between procrastination and sleep quality. We show that the relation is only present for those who have low trait self-control; employees with high trait self-control tend to be immune to low sleep quality. Thus, general advice or interventions to improve sleep quality may be restricted to a selection of employees that are truly affected.

## Introduction

Procrastination is irrational delay that encompasses the discrepancy between intention and action: it occurs when people intend to act but do not act, in spite of knowing that they will be worse off. Many studies have shown that procrastination has detrimental consequences in terms of productivity, health, and well-being (Van Eerde, 2003; Steel, 2007). Not only the delay itself, but also the guilt and shame over the irrational behavior, may impact lives. Ultimately, procrastination may even lead to serious health complaints, depression, and anxiety (Sirois, 2016). Clearly, understanding what factors contribute to this irrational behavior is important (Klingsieck, 2013).

While great advances have been made in unraveling the antecedents and consequences of procrastination (Sirois and Pychyl, 2016), several limitations can be identified in the existing body of work on procrastination. First, while we know relatively much about what predicts procrastination in the academic domain, the procrastination phenomenon has hardly been studied in the work domain. This is rather surprising, given that such self-regulatory behaviors as meeting deadlines and goal achievement are especially compromised by procrastination (Van Eerde, 2003), yet are essential for both individual and organizational performance. Second, procrastination has been studied mainly as a between-person phenomenon, with the assumption that individuals are consistent in their procrastination behavior. However, recent evidence suggests that there is in fact a great deal of daily fluctuation in procrastination behavior (Kühnel et al., 2016, 2017a). This raises the question of which daily predictors of work procrastination are most important. Unfortunately, existing research cannot provide an answer to this question because it has primarily focused on chronic individual differences (e.g., Pychyl and Flett, 2012), which cannot account for within-person fluctuations.

Drawing on the self-regulatory perspective of procrastination (cf. Klingsieck, 2013), which argues that procrastination is a result of diminished self-regulatory resources (Tice and Baumeister, 1997), we argue that within-person antecedents should be sought in daily variables that affect changes in self-regulatory resources. An especially suitable candidate is nightly sleep quality, which has been shown to impact self-regulatory work behavior through its influence on self-regulatory resources (cf. Barnes, 2012) Consistent with this view, recent studies indeed showed that higher daily sleep quality reduces daily work procrastination on the subsequent day (Kühnel et al., 2016, 2017a). Building on this emerging and promising within-person perspective on work procrastination, we argue that a more fine-grained picture of what explains fluctuations in daily work procrastination can be obtained by examining the interplay of within-person sleep quality and between-person antecedents of work procrastination.

In keeping with the self-regulatory perspective of procrastination, we posit that one between-person factor that should play a particularly crucial role in moderating the relation between nightly sleep quality and next-day work procrastination is trait self-control, which refers to the difference between persons in “the ability to override or change one’s inner responses, as well as to interrupt undesired behavioral tendencies (such as impulses) and refrain from acting on them” (Tangney et al., 2004, p. 274), Specifically, as trait self-control can be seen as an employee’s general ability to self-regulate, we expect that the negative impact of nightly sleep quality on next-day work procrastination should be especially pronounced for those low in trait self-control, that is for those who have poor general self-regulatory ability For employees high in trait self-control, this effect should be less pronounced because their ability to self-regulate is already high in the first place.

The contributions of our study are threefold. Firstly, our study contributes to the emerging literature that has started to study procrastination in a work context (e.g., Glazer et al., 2014), a domain in which procrastination should have great significance for both employees and employers. Second, by exploring the dynamic interplay of within-person and static between-person antecedents, our research extends the emerging literature that studies behavior as a combination of these perspectives (cf. Beckmann and Wood, 2017), with a focus on procrastination specifically (e.g., Kühnel et al., 2016). Third, by exploring whether individual differences can modify the significant effects of daily sleep quality on next-day self-regulatory behaviors, we contribute to the literature dealing with the significant role of sleep quality in explaining self-regulatory behavior (e.g., Barber et al., 2017), a research domain which has so far neglected the moderating role of individual differences (Barnes, 2012).

### Procrastination at Work

Procrastination at work has not often been researched (cf. Metin et al., 2016), in contrast to the many studies on procrastination among students. However, work behavior, although different from studying in many respects, also shares some common characteristics with it. Even though procrastination at work has not received much attention, it nevertheless can be assumed that it is an issue of equal, if not higher, prevalence in this domain in comparison to the academic domain. Work is characterized by the omnipresence of activities that demand self-regulation and planning for task completion (cf. Claessens et al., 2010). Furthermore, there is evidence to suggest that students may carry over some of their self-regulatory habits from their study period into their careers (Salmela-Aro et al., 2009).

Procrastination may be seen as avoidance behavior, where the irrational delay serves to regulate negative mood associated with task completion. Avoidant behavior used for mood regulation has been linked to procrastination (Sirois, 2014). Avoidance behavior at work has received attention in the work-related literature, labeled as withdrawal or counterproductive behavior (cf. Van Eerde, 2016). Overall, the literature shows that avoidance behavior at work is linked to diminished well-being and lower performance (Lanaj et al., 2012; Marcus et al., 2016). Procrastination may be considered self-defeating behavior, and is more closely related to withdrawal behaviors than to counterproductive behaviors, as these are usually based on motives against an organization (Marcus et al., 2016). However, procrastination at work is associated with counterproductive work behavior, as well as with boredom and lower engagement (Metin et al., 2016).

### Variation of Procrastination Over Time

Another issue that has not received much attention is the process by which procrastination waxes and wanes over the days. That is, most studies have compared procrastination between individuals, assuming that it is a trait. However, a significant proportion of variance in procrastination can actually be attributed to fluctuations in procrastination from day to day. For example, Kühnel et al. (2016) found that more than half of the variance of procrastination (55%) was within persons. As such, in order to get a complete understanding of work procrastination, it is warranted that researchers start to identify the factors that explain these within-person fluctuations in work procrastination.

### The Impact of Sleep Quality on Self-Regulatory Behavior

One important variable identified as influencing procrastination is sleep quality, as it may influence the energy of a person to engage in work, and to sustain effort over time. When the energy is lacking, procrastination may become more likely. In general, sleep quality may affect performance, health, and attitudes at work (Barnes, 2012; Litwiller et al., 2017). Sleep loss can even become a matter of life and death when bad sleep quality affects safety, and accidents may occur (Åkerstedt et al., 2002; Uehli et al., 2014). Sleep plays an important role in maintaining good health through its replenishing of resources and the recovery associated with it. Restorative sleep implies that a person has no or little difficulties in falling asleep, and no or just a few awakenings during the night. Sleep quality is specifically important. Sleep quantity or sleep duration during the night has not been shown to be related to vitality on a subsequent day (Schmitt et al., 2017), nor to procrastination the next day (Kühnel et al., 2016). The negative relation between sleep quality and procrastination has been shown among students and working adults, in different types of studies, using cross-sectional measurement (Sirois et al., 2015; longitudinally at three points in time over 2 months (Glazer et al., 2014) and in daily diary studies (Kühnel et al., 2016, 2017a).

The importance of sleep has increasingly been recognized in the field of work psychology (Barnes, 2012). From a self-regulatory perspective (e.g., Tice and Baumeister, 1997), many of the issues related to problematic work behavior are related to self-regulation, where cognitive and emotional resources are needed to sustain desirable outcomes at work (Diestel et al., 2015). The impact of bad sleep on work behavior in general is well-described (Litwiller et al., 2017). Not only concentration may suffer, and as a result accidents may occur at work (Åkerstedt et al., 2002), but also work engagement (Kühnel et al., 2017b). Other outcomes that have been shown to be related to sleep are workplace deviance (Christian and Ellis, 2011) and job satisfaction (Scott and Judge, 2006).

Besides having energy available for action, having slept well may also help in overriding impulses. Sleep is critical to self-regulation (Barnes, 2012), and self-regulation impairment is related to poor sleep (e.g., Wagner et al., 2012). Self-regulation is assumed to be taxing, both cognitively or emotionally. When sleep quality is compromised, people may feel too tired to overcome problems in self-regulation, such as resisting temptations.

Sleep helps to replenish self-regulatory resources; fatigue may break down the strength needed for self-regulation. Having self-regulatory resources available should help to overcome procrastination: more resources are available to concentrate, persist, and motivate oneself (e.g., Lanaj et al., 2014). That is, when enough energy is available, giving in to distractions, unwanted intrusions and spontaneous behaviors that do not serve the person’s longer term goals may be resisted, resulting in better self-regulation. Procrastination means that a person delays and does not initiate and persist on actions. Having energetic and self-regulatory resources available will help the person to initiate action and to remain focused. As such, feeling replenished and recovered, and having enough energy may be a precondition to prevent procrastination because of increased resources (Gröpel and Steel, 2008). Overall, having sufficient energy available may serve both as a precondition for action and as a strength for self-regulation to stay on the intended course of action.

Several studies focused on sleep and work outcomes on the next day, such as engagement at work (Kühnel et al., 2017b); unethical conduct as assessed by supervisors (Barnes et al., 2011); vigor (Clinton et al., 2017); and proactive behavior (Schmitt et al., 2017). As far as we know there are two studies that investigated the role of sleep on procrastination in a diary study (Kühnel et al., 2016, 2017a). All studies confirm the important role of sleep quality during the night for behavior the next day. High sleep quality increases the availability of limited resources such that these can be directed at work activities that need self-regulation rather than avoiding them by procrastination. Consistent with this theoretical and empirical knowledge, we expect a negative relation between sleep quality and procrastination.

Hypothesis 1: Within persons, nightly sleep quality will be negatively related to work procrastination the next day.

So far we discussed and reflected on the power of nightly sleep quality as a variable in explaining variations in within-person work behavior, including work procrastination, an empirical reality that would speak to the importance of focusing on increasing employee sleep quality. Given the consistently demonstrated impact daily sleep quality has on self-regulatory behavior the subsequent day, it may be tempting, at first glance, to generalize such effects to every employee. The question remains, however, to what extent the significant impact of sleep quality on work procrastination holds for every employee. Are some employees more affected by fluctuations in sleep quality than others? Framed differently, and perhaps more interestingly, are there employees who are relatively unaffected by or immune to the powerful effects of nightly sleep quality on subsequent daily work procrastination? We are not aware of any studies addressing this pertinent issue of individual differences in self-regulation with regard to daily procrastination. Previous studies addressed time-related issues as person-level moderators of the relation between sleep quality and daily procrastination, such as social jet lag (Kühnel et al., 2016) and the match between chronotype and shiftwork (Kühnel et al., 2017a).

Similarly, in the sleep literature, Barnes (2012) noted that most of the sleep literature to date has focused on main effects of sleep on outcomes at work and that only a few studies have considered the potential of individual differences as moderators of such effects. Demographic variables such as age and gender may play a role. For example, Scott and Judge (2006) found that women were more affected by a poor night of sleep than were men on outcomes such as fatigue, attention, and joviality. Barnes suggested the differences should be treated with caution, and emphasized the need for sound theoretical explanations for moderation. He maintained that only extraversion and conscientiousness made sense theoretically as moderators of the relation between sleep quality and work outcomes. In line with this reasoning, one study showed that extraverted individuals are more vulnerable to sleep deprivation for staying alert because of to their higher need for stimulation (Killgore et al., 2007). Moreover, Wagner et al. (2012) reasoned that discipline may be important and found that individuals’ sleep interruption affected cyberloafing the next day much more for low than for high conscientious people.

Taken together, research on between-person moderators of the relation between daily sleep quality and daily work behavior remains scarce, especially with regard to daily work procrastination as outcome variable. Moreover, given that self-regulatory resources play a center role in explaining the relation between sleep quality and work behavior that involves self-regulatory behavior (Diestel et al., 2015), we find it remarkable that between-person moderators related to self-regulation have not been considered so far. Therefore, answering Barnes’ (2012) call for more research on both different moderators for different types of sleep-effects and theoretically sound moderators, we advance trait self-control as a suitable candidate in this regard. This variable is specifically focused on such self-regulatory capacities as resisting temptations and inhibiting unwanted impulses.

### Daily Sleep Quality, Procrastination, and Trait Self-Control

Self-control is defined as “the ability to override or change one’s inner responses, as well as to interrupt undesired behavioral tendencies (such as impulses) and refrain from acting on them” (Tangney et al., 2004, p. 274), and thus can be seen as general self-regulatory ability. Trait self-control can be referred to as the differences between persons in this ability. A comprehensive review of the role of self-control at work is provided by Lian et al. (2017), showing the relevance in the work context.

Given its relation with self-regulation, trait self-control should play an important moderating role in the relation between sleep quality and daily work procrastination. Recall that higher nightly sleep quality through its impact on self-regulatory resources should decrease work procrastination the subsequent workday. Because trait self-control refers to the between-person difference in the ability to self-regulate, the self-regulatory-enhancing effects of better sleep quality should be beneficial especially for employees who have poor self-regulatory abilities, that is, those who score low on trait self-control (Diestel et al., 2015). On the other hand, the energy-depleting or increasing effects of variations in sleep quality should have a weaker impact on employees high on self-control, because these individuals have the trait-like ability to self-regulate, which likely functions as a natural buffer. Put differently, it can be expected that self-control has a diminishing effect on the relation between sleep and procrastination, such that the negative relation between sleep quality on procrastination is less pronounced for those who are high on self-control.

*Hypothesis 2: The within-person relation between nightly sleep quality and work procrastination the next day will be moderated by trait self-control, such that this negative relation will be weaker when trait self-control is high* (*vs. weak*).

## Materials and Methods

### Participants and Procedure

The sample consisted of employees who were recruited by students working on a research project. These students approached working adults in their own network, who in turn were asked to approach other potentially interested working adults. The research was approved by the Ethics Committee in the school. A total of 71 participants completed an initial, one-time questionnaire during the first wave, and then two or more sets of morning and afternoon surveys during the second wave, representing a response rate of 81%. Employees consisted of 51% males, had an average age of 35.20 years (*SD* = 12.74), had an average employment tenure of 13.3 years (*SD* = 13.16), and worked on average 41.94 h per week (*SD* = 6.99). The sample comprised a diverse range of professions within industries including finance or banking (17%), government or education (13%), construction (7%), health care (7%), sales or marketing (6%), and others (e.g., legal, security, and unreported).

Data were collected in two waves. During the first wave we assessed trait self-control with one survey. One week after the first wave, morning and afternoon surveys were administered daily for 10 consecutive workdays during the second wave. We sent a link to respondents for the online morning survey around 11 AM and for the afternoon survey around 4 PM each workday. Nightly sleep quality (of the night before) was assessed in the morning survey, and daily work procrastination was assessed on the afternoon survey at the end of the workday. Individuals did not complete surveys if they were absent from work, for example due to sick leave. Even though all focal variables were assessed by the same person, concerns of common source bias were reduced, because the level-1 (daily) variables were measured at different times (Podsakoff et al., 2003). This temporal separation of measurements also increases confidence in establishing causal precedence between predictor and outcome (Brewer, 2000).

Ultimately, we obtained a total of 465 of the possible 710 matched morning and afternoon surveys, representing a 65% response rate at the daily level. Thus, the final sample consisted of 465 matched level-1 (within-person) observations and 71 level-2 (between-person) observations.

### Measures

Following the recommendations of Uy et al. (2010) and Beal (2015), we shortened previously validated scales to encourage high response rates and lessen participant fatigue. This approach is commonly used in daily experience sampling studies generally (e.g., Johnson et al., 2012) and daily experience sampling studies involving work procrastination specifically (e.g., Kühnel et al., 2016). Coefficient alphas for the level-1 variable work procrastination were averaged across the days of data collection.

#### Trait Self-Control

We measured trait self-control on the one-time survey using four items (α = 0.76) developed by Tangney et al. (2004) and successfully adapted by Smit and Barber (2016). The items are: “I am good at resisting temptation,” “I have a hard time breaking bad habits (reverse coded),” “I wish I had more self-discipline (reverse coded),” and “people would say I have iron self-discipline.” Participants responded to these items using a five-point scale (from 1 = “Strongly disagree” to 5 = “Strongly agree”).

#### Nightly Sleep Quality

Sleep quality was assessed with a single item adapted from the Pittsburgh Sleep Quality Index (Buysse et al., 1989). The item read “How would you evaluate last night’s sleep?” and was rated on a five-point scale (from 1 = “Very bad” to 5 = “Very good”). This approach of assessing sleep quality has been employed successfully in previous experience sampling studies (e.g., Sonnentag et al., 2008; Hülsheger et al., 2014; Kühnel et al., 2016).

#### Daily Work Procrastination

Work procrastination was measured at the end of the workday using the three highest loading items (average α = 0.91) from the procrastination scale developed by Tuckman (1991) and adapted by others in a work context (e.g., Kühnel et al., 2016). The items are: “Today, I was an incurable time waster,” “Today, I was a time waster, but I couldn’t seem to do anything about it,” and “Today, I promised myself I would do something and then dragged my feet.” Participants responded to these items using a five-point scale (from 1 = “Completely disagree” to 5 = “Completely agree”).

## Results

Given the nested structure of our data we conducted multilevel analyses using the multilevel package in R (Bliese, 2013). Sleep quality and procrastination are the level-1 variables, and trait self-control is the level-2 variable. As per recommendations by Hofmann et al. (2000), the level-1 predictor (sleep quality) was person-mean centered and the level-2 variable (trait self-control) was grand-mean centered. By person-mean centering the level-1 predictor, we ensure that between-person differences cannot account for any observed covariance between level-1 variables (Bryk and Raudenbush, 2002).

Justifying the use of multilevel analyses to test our hypotheses, of the total variance in sleep quality and procrastination 77 and 64% was at the within-person level, respectively. Table 1 provides the descriptive statistics and the bivariate correlation for the variables in the study. There are three variables in the study: one measured at the between-person level (self-control) and two at the within-person level (sleep quality and procrastination). The correlations above the diagonal represent the aggregated scores for sleep quality and procrastination at the between-person level.

**Table 1:** Descriptive statistics and correlations.

	*M*	*SD*	*r*		
			1	2	3
(1) Self-control	3.18	0.71		0.21	-0.30^∗∗^
(2) Sleep quality	3.58	0.84	–		-0.38^∗∗∗^
(3) Procrastination	2.01	0.78	–	-0.23^∗^	

*Person level correlations are given above the diagonal *n* = 71. Day level correlations are given below the diagonal, *n* = 465. ^∗^*p* < 0.05, ^∗∗^*p* < 0.01, ^∗∗∗^*p* < 0.001*.

**FIGURE 1:**
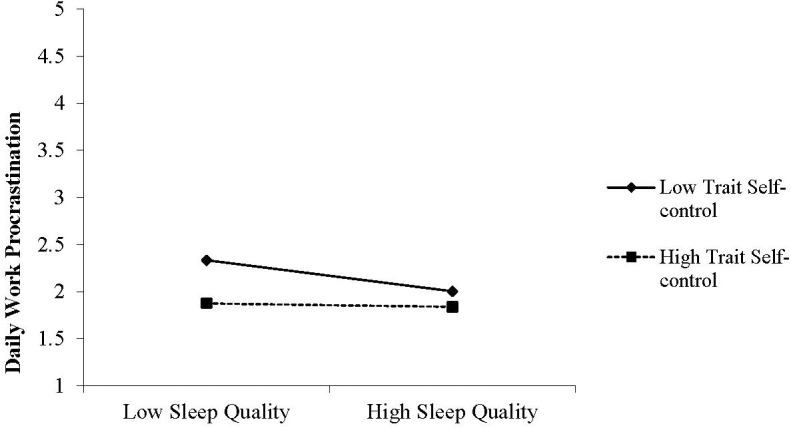
The relation between sleep quality and daily procrastination moderated by trait self-control.

Hypothesis 1 predicted that higher sleep quality would be associated with lower procrastination on the subsequent workday. Multilevel analysis revealed that this relationship was statistically significant (γ = -0.14, *t* = -3.00, *p* = 0.00), thus providing support for Hypothesis 1. Hypothesis 2 predicted that the daily within-person relationship between sleep quality and procrastination during the subsequent workday would be moderated by trait self-control, such that the within-person relationship between sleep quality and procrastination would be weaker for persons high in trait self-control. Cross-level moderation analysis revealed that the cross-level moderating effect of trait self-control was significant (γ = 0.15, *t* = 2.74, *p* = 0.01). Simple slope tests (Aiken and West, 1991) indicated that the effect of sleep quality on daily procrastination was negative and significant when trait self-control was low (γ = 0.23, *t* = -4.33, *p* = 0.00), whereas this effect was not significant when trait self-control was high (γ = -0.02, *t* = -0.24, *p* = 0.81). We conclude that Hypothesis 2 was supported (see Figure 1). The full model explained 6% of the variance in daily procrastination. Effect sizes in mixed models are not as straightforward as in regression models, and there is no consensus among scholars regarding the most appropriate measure (Peugh, 2010; Aguinis et al., 2013). One measure that comes closest to the traditional change in explained variance in the outcome (Δ R^2^ in regression models) is the pseudo R^2^ in mixed models. In our model, the interaction term explains an additional 3% of variance in procrastination, an effect that is common in field studies in this area. In addition to such a global measure, the coefficients may be interpreted as local effect sizes. The coefficients are unstandardized. An example of the interpretation would be for the effect of sleep quality on procrastination at low levels of self-control: a daily increase in sleep quality of one-point (on a five-point scale) is associated with a change in daily work procrastination of 5% on a five-point scale (or 29.5% in standard deviations, or 38% in terms of the total variance in daily procrastination). While it is difficult to interpret what this means in terms of manifest procrastination, we believe that it represents a meaningful impact for employees at work. See for a further interpretation of effect sizes in multilevel models Peugh (2010) and Aguinis et al. (2013).

Finally, we performed a supplementary analysis to test the robustness of our findings. Although we showed that sleep quality as assessed in the morning predicted work procrastination in the afternoon, a more conservative test involves testing the respective effect after controlling for previous levels of procrastination, enabling us to test changes in work procrastination. Thus, we re-ran a multilevel analysis but this time controlling for both sleep quality assessed the prior day (i.e., sleep quality of the night that preceded the prior workday) and work procrastination assessed the prior day. Such a test enabled us to demonstrate whether a change in sleep quality predicts a change in work procrastination. Results, which were based on 344 level-1 observations and 68 level-2 observations, revealed a significant main effect of nightly sleep quality (γ = -0.13, *t* = -2.52, *p* = 0.02). More importantly, the interaction effect again turned out to be significant (γ = 0.13, *t* = 2.06, *p* = 0.01). The nature of this interaction was identical to that of the original results described in the previous section. All in all, these results speak to the robustness of our findings and to the causal direction of our theorized relationship.

## Discussion

As hypothesized and consistent with previous diary studies (Kühnel et al., 2016, 2017a), we found a relation between sleep quality and procrastination the next day. Additionally, we hypothesized and found the moderating effect of trait self-control. Not only was the relation between sleep quality and procrastination lower for those with high versus low self-control, it was even non-significant for respondents high on self-control. This implies that sleep quality is more important for those low on self-control, as only for these respondents, it was negatively related to procrastination the next day.

Our study makes three contributions to the literature. First, we contribute to the limited number of studies of procrastination in the work domain, where most studies have been conducted on academic procrastination. The work domain, however, given its emphasis on autonomy in reaching deadlines, provides a relevant setting for studying procrastination from a self-regulatory perspective. Second, we add to the studies on the dynamics of daily procrastination by showing that a large part of the variance (64%) in procrastination is explained at the daily level, showing that a much larger proportion of the behavior may be explained by within rather than between person variables. In other words, procrastination is not consistently high or low over the days but fluctuates over time. This underscores the relevance of studying more within-person antecedents of procrastination. Furthermore, by examining the interplay between within-person sleep quality and procrastination, and the between-level moderator self-control, we extend this emerging literature by showing the effect of individual differences in self-control on the daily fluctuations in procrastination: the relation is only negative for those who are low on self-control. For those high on self-control, there is no relation between daily sleep quality and procrastination. Apparently, this personality trait helps to curb procrastination even if sleep quality is low. This demonstrates the value-added from including between-level moderators into the equation (see also Kühnel et al., 2016, 2017a), and warrants the need to adopt a similar model in future research as well. Third, we add to the literature on the importance of sleep to procrastination and work behavior. Sleep quality has been shown to affect many outcomes at work. Some studies have focused on the dynamics of sleep quality at the daily level, but only a limited number of studies has investigated sleep quality and daily procrastination.

Our study found that not only the variance in procrastination at the within-person level was high, indicating the variability of procrastination over days and the negative relation with sleep quality of the previous night, but also found a strong effect of the differences in self-control between persons. Even though we expected a stronger negative relation between sleep quality and procrastination for those low on self-control, we still expected an effect for those high on self-control as well, as the relation between sleep and procrastination would appear to apply to everyone. However, the relation between sleep quality and procrastination the next day was absent for those high on self-control. The mediating mechanism assumed was that feeling more rested helps to overcome procrastination. All respondents may experience the effects of sleep quality, but only for those who are low on self-control there is a relation with procrastination. This raises the question which alternative mediating mechanisms played a role. We will address these below in our suggestions for future research.

A potential limitation of our study is that it was based on self-reported variables only. While sleep quality and procrastination are best assessed by the use of self-reports, the measurement of variables by means of self-reports is subject to common method bias (Podsakoff et al., 2003). However, concerns of common method bias were minimized in several ways. First, we ensured an interval between the measurement of the focal predictor and the assessment of the dependent variable, one of the best methods to minimize common source bias if no other sources are used (Podsakoff et al., 2003). Second, the level-1 (daily) predictor sleep quality was mean-centered before data-analysis, alleviating the possibility that between-person differences in response tendencies would account for the results – all daily results are relative to the person’s mean score. Third, common method bias tends to deflate interaction effects (Siemsen et al., 2010). As our results revealed a significant interaction effect, common method cannot account for this result. Altogether, we are confident that the validity of our conclusions has not been compromised by the use of self-reports. Nevertheless, despite our confidence in the veracity of the results, we encourage researchers to test and replicate these findings in different samples and domains.

Future research directions may be taken at both levels of analysis. First, the issue of mediators raised above may need more research attention, in order to investigate the effect of sleep quality further. At the within-person level, these may include daily events or states – to elucidate the findings further. It remains unclear why high self-control employees remain unaffected by changes in sleep quality. Our assumption was the ability to self-regulate would equip these individuals with a buffer to possible resource-depleting effects of poor sleep quality. This suggests that these individuals would not experience a depleted state after poor nightly sleep quality. Even so, we cannot be conclusive about this issue. An alternative explanation is that these individuals do experience depleted resources, just like their counterparts low on self-control, but remain unaffected by it. For example, because they rely on self-efficacy derived from their perceived self-regulatory abilities. As such, these individuals may experience depleted resources, but this does not make them vulnerable to procrastination because their self-perceived self-regulatory ability causes them to have confidence in their ability to complete their goals and tasks. In order to test these ideas, future research may include self-efficacy or state self-control as within-person mediators.

Second, existing studies adopting the within-person perspective focus on sleep quality, which makes sense given its relation to self-regulatory capacity. However, much remains unknown about the predictors of procrastination at the daily level. Other predictors than sleep quality may be investigated in future research to establish the effects within the self-regulation framework on procrastination, such as failure, frustration, or other negative emotional events at earlier points in time. Other predictors may include positive or energizing events, variables, such as breaks, successes, meditation, and positive social interactions.

Finally, future research directions at the between-person level may incorporate other moderators within the self-regulation framework. Self-control was an obvious candidate in explaining how people deal with the effects of sleep quality, but other variables that help to explain how depletion is dealt with may also be investigated. For example, the Big Five factor conscientiousness has not been researched as a moderator of the within-relation between sleep quality and procrastination yet. Other traits that may be tested as moderators are impulsivity and neuroticism.

The results have practical implications for procrastination interventions (cf. Van Eerde and Klingsieck, 2018), in that a more personalized version of an intervention may be developed the more is known about which antecedents, both at the daily and the person level, may affect the occurrence of procrastination. In general, “energizing respite” interventions (Steidle et al., 2017) may help to build energy at work, but their effect on procrastination has not been researched, nor have daily intervals been the unit of analysis. With regard to training self-control, much is unknown; a meta-analysis did not show convincingly that repeated training of self-control resulted in a higher level of self-control (Friese et al., 2017).

The results may also have implications for sleep interventions. Training healthy sleep habits may have more impact on those low on self-control. Insomnia interventions were shown to lead to increased self-control (Barnes et al., 2017), suggesting that state-like features of self-control may be trained using sleep interventions. Individual differences were not taken into account in Barnes et al. (2017), but they may be highly relevant with respect to who to treat and how intensive treatment should be.

Overall, our study finds support for the hypotheses based on the self-regulation framework of procrastination, incorporating daily measurements of sleep quality and procrastination. We show that both sleep quality and procrastination are variable over the days, and are negatively related, but also that their relation depends on individual differences in self-control. Our findings contribute to theory on procrastination and sleep, and have implications for practice in helping people at work wishing to curb their procrastination.

## Ethics Statement

The research was approved by the Ethics Committee Economics and Business (EBEC) of the University of Amsterdam. The consent of the participants was implied through survey completion after they were provided with all necessary information.

## Author Contributions

All authors listed have made a substantial, direct and intellectual contribution to the work, and approved it for publication.

## Conflict of Interest Statement

The authors declare that the research was conducted in the absence of any commercial or financial relationships that could be construed as a potential conflict of interest.
